# Microvascular changes in the recurrent cystoid macular edema secondary to posterior noninfectious uveitis on optical coherence tomography angiography

**DOI:** 10.1007/s10792-022-02327-0

**Published:** 2022-05-22

**Authors:** Valeria Albano, Silvana Guerriero, Claudio Furino, Giancarlo Sborgia, Alessandra Sborgia, Rosanna Dammacco, Francesco Boscia, Giovanni Alessio

**Affiliations:** Department of Basic Medical Sciences, Neurosciences and Sense Organs, Institute of Ophthalmology, University of Bari, Piazza Giulio Cesare 11, 70124 Bari, Italy

**Keywords:** Non-infectious posterior uveitis, Cystoid macular edema, Optical coherence tomography angiography

## Abstract

**Background:**

Posterior uveitis represents the second most frequent type of uveitis (15–30% of all uveitis). Noninfectious posterior uveitis complicated with secondary cystoid macular edema (CME) affects the visual prognosis negatively. The objective of the current study is to detect possible microvascular changes causing relapsing uveitis-related CME using optical coherence tomography angiography (OCTA).

**Methods:**

This is an interventional, observational, retrospective study with 1 year follow-up. Patients with noninfectious, posterior uveitis-related CME undergoing dexamethasone (DEX) implant were evaluated. Following the DEX-implant were carried out control visits after 1 month, 2-months, 4-months, 6-months, and for up 1-year. A total of 76 eyes of 38 consecutive patients with noninfectious posterior uveitis were enrolled (consecutive sample). Complicated noninfectious posterior uveitis with secondary CME was diagnosed in 56 eyes of uveitis patients (73.7%) and reviewed.

**Results:**

Our investigation showed (1) a reduction in superficial vessel plexus (SVP) measurements within 2-month (84%), reaching 96.4% for up 1-year, (2) an irregular profile of SVP in 69.6% of cases, persisting for up 1-year; relapsing uveitis-related CME eyes with irregular superficial foveal avascular zone (FAZ) profile were in 51%, while the SVP measurements reestablished in 100% of cases. Conversely, (3) the deep vascular plexus (DVP) parameters restored in a lower number of eyes within the 2-month (39.3%), remaining abnormal in 46.4% of cases for up 1-year; despite DVP restored in 53.6% of cases for up 1 year, (4) a capillary rarefaction ring around the FAZ appeared in 80.4% of cases; the relapsing uveitis-related CME eyes with abnormal DVP parameters were present in 41% of cases, of which 92.1% showed a rarefaction ring had abnormal DVP.

**Conclusions:**

The use of OCTA enabled the evaluation in detail of retinal microvascular changes. We suggested that the possibility of the recurrence of the uveitis-related CME depends on the persistence of modifications of the superficial and deep layers. In this regard, we propose to implement the current imaging armamentarium with OCTA for the follow-up of patients with noninfectious uveitis-related CME.

## Introduction

Posterior uveitis represents the second most frequent type of uveitis (15–30% of all uveitis) [1]. These forms cause unilateral visual impairment in 14–50% of cases, and bilateral in 4–40% [2]. Posterior uveitis are infectious or noninfectious etiology. Noninfectious posterior uveitis includes several entities that can be associated with autoinflammatory, or autoimmune diseases [3], affecting the retina, and choroid, sometimes involving adjacent structures, such as the vitreous, optic nerve [4]. The treatment with systemic steroid, or immunosuppressive therapy, depends on the underlying disease [5]. In some cases, noninfectious posterior uveitis is complicated with secondary cystoid macular edema (CME), estimated in various studies about 20–70%, negatively affecting the visual prognosis [6]. CME in uveitis depends on the intraretinal accumulation of fluid, due to the alteration of the integrity of the blood-retinal barrier (BRB) [7]. If the inflammatory stimulus is fleeting, then BRB restores spontaneously, while if it enduring, focal or diffuse leakage occurring in the extracellular space of the retina, mainly at the level of the external plexiform layer (layer of Henle) [8]. A negative correlation between CME, macular thickness, and visual acuity was revealed in the study by Iannetti et al. [9]; the positive correlation between CME and duration of uveitis was also described [10]. CME was studied employing imaging techniques commonly used, such as fluorangiography (FA), indocyanine green angiography (ICG-A), and optical coherence tomography (OCT) [11]. Latest technological developments have led to the innovative introduction of optical coherence tomography with angiography modules (OCT-A), which accurately detecting ultrastructural details of the retinal capillaries, not otherwise identified [12].

For the treatment of CME related to noninfectious posterior uveitis, the biodegradable dexamethasone 0.7 mg with the intravitreal implant has been approved by the US Food and Drug Administration (US-FDA) in 2010 [13], and by NICE (and Care Excellence National Institute) in 2017 [14, 15] for the treatment of the persistent noninfectious uveitis-related CME [5]. Previous studies have investigated the benefits and limits of dexamethasone implantation (DEX-implant) in uveitis were either prospective or retrospective [16–19]. Since the trial by Lowder et al. [20], the safety, tolerability, and efficacy of the DEX-implant in noninfectious uveitic macular edema (ME) was reported [21–24]. However, the treatment was not always lasting in the long-term, and some cases of recurrent CME associated with uveitis were recorded [25]. The pathogenesis of the recurrence of CME in uveitis may be anatomical and functional changes of the retinal vessels [26]. This study aims to evaluate the microvascular changes following the DEX-implant in patients who presented secondary CME noninfectious posterior uveitis-related.

## Methods study design

This is an interventional, observational, retrospective study with 1 year follow-up, an evaluation of patients with secondary CME noninfectious posterior uveitis-related undergoing DEX-implant. The current article does not contain any personal information that could identify the patient. It adhered to the tenets of the Declaration of Helsinki. All participants signed the informed consent before the surgery. The Standards for Reporting Diagnostic Accuracy (STARD) statement was developed [27].

### Participants

From January 2020 to December 2020 a total of 76 eyes of 38 consecutive patients with noninfectious posterior uveitis referred to Uveitis University Ophthalmology Center of the Bari Polyclinic were selected. Complicated noninfectious posterior uveitis with secondary CME was diagnosed in 56 eyes of uveitis patients (73.7%) and reviewed. All patients with uveitis-related CME were treated with a single shot of DEX-implant. The age range of the sample was 24–84 years (mean 54 ± 42.4 years). The inclusion criteria were (1) confirmed diagnosis of uni- or bilateral noninfectious posterior uveitis, (2) new referral to Uveitis University Ophthalmology Center of the Bari Polyclinic, (3) the presence of the secondary CME of recent onset confirmed by the OCT findings, and (4) CME not previously treated with intravitreal drugs. All patients who met the criteria were included. Conversely, study exclusion criteria were (1) noninfectious posterior uveitis without related secondary edema, (2) previous intravitreal DEX-implant, (3) previously intravitreal injections of other substances, (4) ocular hypertension, (5) presence of serous retinal detachment, and (6) previous retinal intraocular surgery. Demographic and anatomical characteristics of the study sample are summarized in Tables 1 and 2. Patients were previously treated with systemic therapy, immunosuppressive drugs (methotrexate, or azathioprine), or corticosteroids, depending on the underlying disease. None of patients were on biological therapy. In all those eyes in which CME was diagnosed they received a sustained-release 0.7 mg intravitreal DEX-implant (DEX-implant, Ozurdex®, Allergan, Inc.). All patients were followed for 1 year.

**Table 1:** Demographic characteristics of the study sample

	Sample size (*n* = 38 participants)	*P* value
*Age (y, range)*		0.05
20–35, *n* (%)	3 (7.9)	
36–50, *n* (%)	17 (44.7)	
51–60, *n* (%)	16 (42.1)	
≥ 60, *n* (%)	2 (5.3)	
*Gender*		0.22
Female, *n* (%)	18 (47.4)	
Male, *n* (%)	20 (52.6)	

*Y* years, *N* number, % percent

**Table 2:** Anatomic characteristics of the 56 eyes included

	Sample size (*n* = 56 eyes)	*P* value
*Eyes*		0.18
Right, *n* (%)	24 (42.9)	
Left, *n* (%)	32 (57.1)	
*Laterality*		0.04
Unilateral, *n* (%)	12 (31.8)	
Bilateral, *n* (%)	22 (57.9)	
*State of eye*		0.08
Phakic, *n* (%)	31 (55.4)	
Pseudophakic, *n* (%)	25 (44.6)	
*Course of uveitis*		0.06
Acute, *n* (%)	8 (21)	
Recurrent, *n* (%)	18 (47.4)	
Chronic, *n* (%)	12 (31.6)	
*Etiology of uveitis*		0.06
Uveitis not associated with systemic disease, *n* (%)	8 (21)	
Uveitis associated with systemic disease, *n* (%)	18 (47.4)	
Idiopathic uveitis, *n* (%)	12 (31.6)	
*Previous systemic treatment*		0.07
Corticosteroids, *n* (%)	25 (65.8)	
Immunosuppressors, *n* (%)	13 (34.2)	
*BCVA (logMAR) at inclusion, range*		0.04
< 0.1, *n* (%)	4 (7.1)	
0.1–0.4, *n* (%)	15 (26.8)	
0.5–1.0, *n* (%)	28 (50)	
> 1.0, *n* (%)	9 (16.1)	
*IOP (mmHg) at inclusion*		0.18
8–14	32 (57.1)	
15–21	24 (42.9)	
*OCT macular findings*		0.06
CME with the epiretinal membrane, *n* (%)	20 (35.7)	
CME without the epiretinal membrane, *n* (%)	36 (64.3)	
*Recurrent CME during the follow-up*		
M1	0	
M2	0	
M4	21 (37.5%)	
M6	9 (16.1%)	
M12	0	

*N* number, *%* percent, *BCVA* best corrected visual acuity, *IOP* intraocular pressure, *OCT* optical coherence tomography, *CME* cystoid macular edema, *M1* 1 month, *M2* 2-month, *M4* 4-month, *M6* 6-month, *M12* 12-month

### Clinical examination

**Fig. 1:**
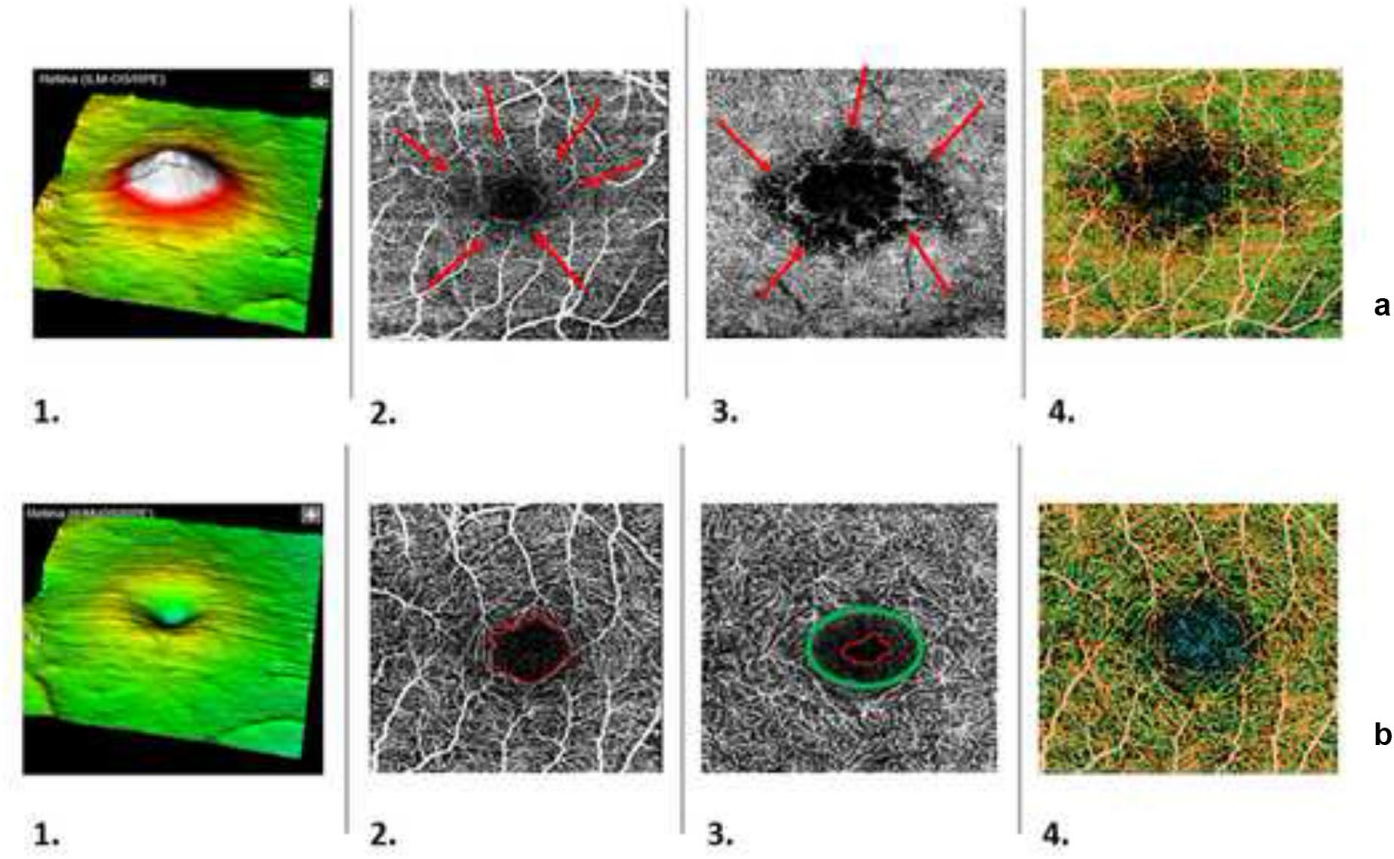
Representative images on a uveitis-CME eye of the sample study before the DEX-implant treatment (**a**) and after follow-up 6-month (**b**) showed. (1) The 3D map of the CMT (SD-OCT) (2) The SVP scan centered on the FAZ (SS-OCTA). (3) The DVP scan centered on the FAZ (SS-OCTA). (4) En face image with montage scanning protocol (SS-OCTA). The increase of the CMT before the DEX-implant (**a,1**) and the reduction of the CMT after the DEX-implant (**b,1**) are illustrated. The cystic fluid is outlined by red arrows around the FAZ area in the SVP (**a,2**) and DVP (**a,3**) layers. The irregular FAZ profile in the SVP (**b,2**) and DVP (**b,3**) layers are marked in the red line. The capillary rarefaction ring in the DVP network is pointed out with a green circle (**b,3**)

The visits following the DEX-implant were carried out after 1 month (M1), 2-months (M2), 4-months (M4), 6-months (M6), and for up 1-year (Y1). Examinations were performed as following: the best-corrected visual acuity (BCVA, logMAR), in vivo biomicroscopy, measurement of intraocular pressure (IOP, mmHg) using Perkins applanation tonometer, spectral domain optical coherence tomography (SD-OCT, RTVue XR Spectral Domain OCT, Optovue Inc, Fremont, USA), optical coherence tomography angiography (OCT-A, SS OCT Angio ™ into Swept Source DRI OCT Triton ™, Topcon Medical Systems, Inc.). The central macular thickness (CMT) was indagated through SD-OCT by MM6 scanning. The foveal avascular zone (FAZ), the superficial vessel plexus (SVP), and the deep vessel plexus (DVP) were examined by OCTA data analysis into a 3 × 3 mm^2^ parafoveal window, through a split-spectrum amplitude de-correlation algorithm (SSADA) [28] (see Fig. 1). FAZ area was set manually.

### FAZ area measurements

FAZ area outlined after importing image records to Adobe Photoshop (Adobe Photoshop CC 2018 (19.0), Adobe Systems, San José, California, USA) as JPEG-file. The borders of FAZ were defined in red color. Area quantification was also performed in Adobe Photoshop.

### Statistical analyses

The statistical analyses were performed using SPSS Statistics for Windows, version 23.0 (SPSS Inc., Chicago, Ill., USA). Comparisons between groups were performed using the nonparametric Mann–Whitney *U*-test. Categorical comparison was made using a Pearson’s Chi-squared test. We have assumed statistical significance at *p* < 0.05.

## Results

A significant improvement in BCVA during the 1-year follow-up from T0 (time of inclusion) to 1Y (mean 0.3 ± 0.2 logMAR, range 0.8–0.1 logMAR, *p* = 0.001) has been recorded. There were no significant increases in IOP up the 1-year following the DEX implantation (mean 14.5 mmHg ± 9.2, range 8–21 mmHg, *p* = 0.006). OCTA data are shown in Table 3. In the sample size examined, the mean CMT was decreased from baseline (514.96 ± 141.88 µm) to the 1-year follow-up (245.65 ± 143.81 µm), range 215.43–650.14 µm (*p* = 0.001). The CME recovery (as a complete resolution of fluid) was established after DEX implant, as following: in 27 eyes (48.2%) the CME at M1, in 15 eyes (26.8%) at M2, in 6 eyes (10.7%) at M4, in 5 eyes (8.9%) at M6, in 3 eyes (5.4%) at Y1. CME recurred in 30 eyes (53.6%) treated with one single shot of DEX implant; of these 18 (32.1%) had an ERM at inclusion. The relapsing CME occurred at M4 in 21 eyes (37.5%), and M6 in 9 eyes (16.1%). All of these relapsed eyes replanted as soon as CME reappeared. At 1 year, 28 eyes (93.3%) had a complete resorption, in the absence of a recurrence of uveitis. None of the patients were on antiglaucoma medications at the end of the study.

**Table 3:** OCT and OCTA data of the study sample during follow-up

	T0	M1	M2	M4	M6	Y1
CMT, mean (SD), µm	514.96 (141.87)	329.85 (157.43)	326.75 (123.01)	331.07 (141.56)	332.78 1 (171.06)	245.56 (124.78)
SVP, mean (SD), mm^2^	1.01 (0.28)	0.55 (0.39)	0.54 (0.34)	0.52 (0.28)	0.51 (0.36)	0.31 (0.22)
DVP, mean (SD), mm^2^	0.71	0.55 (0.16)	0.52 (0.18)	0.51 (0.19)	0.51 (0.18)	0.48 (0.15)

*CMT* central macular thickness, *SVP* superficial vessel plexus, *DVP* deep vessel plexus, *T0* inclusion, *M1* 1 month, *M2* 2-month, *M4* 4-month, *M6* 6-month, *Y1* 1 year

### Superficial, and deep FAZ area changes

OCTA data are shown in Table 3. The enlargement of the FAZ in the SVP was restored, as following: in 32 eyes (57.1%) at M1, in 15 eyes at M2 (26.9%), in 3 eyes at M4 (5.3%), 3 eyes (5.3%) at M6, ad in 1 eye (1.8%) at 1Y; in 2 eyes (3.6%) it remained enlarged despite the DEX implant. The mean superficial FAZ area was significatively reduced from baseline 1 ± 0.28 mm^2^ to 0.31 ± 0.22 mm^2^ at the end of the 1-year follow-up (range 0.12–1.23 mm^2^, *p* = 0.001). The FAZ area in SVP was irregular in 39 eyes (69.6%) at the end of 1-year follow-up The FAZ diameter was reestablished in DVP, as following: in 10 eyes (17.9%) at M1, in 12 eyes (21.4%) at M2, in 4 eyes (7.1%) at M4, in 2 eyes (3.6%) at M6, and in 2 eyes at 1Y (3.6%); in 26 eyes (46.4%) it remained enlarged despite the DEX implant. The mean deep FAZ area was not significatively reduced from baseline 0.71 ± 0.17 mm^2^ to 0.48 ± 0.15 mm^2^ at the end of the 1-year follow-up (range 0.25–0.86, *p* = 0.001). In the deep FAZ, a capillaries rarefaction appeared around the FAZ in 45 eyes (80.4%).

## Discussion

One of the most common complications of noninfectious posterior uveitis is CME [29]. Markomichelakis et al. have identified two main patterns of ME, with no statistical significance in relation to the location, or etiology of uveitis: (1) diffuse type (DME), and (2) cystoid type (CME) [30]. The incidence of CME has been estimated in various studies about 33% of uveitis patients [30]. In recent reports, the use of OCT has revealed the CME type in 25–69% of patients with uveitic ME examined [9, 30]. In essence, the presence of CME was observed especially in higher age of patients at the onset of uveitis, insidious onset of uveitis, persistent duration of an attack of uveitis, a chronic course of uveitis, bilateral involvement. In accord with previous studies [31–33], the occurrence of CME in noninfectious posterior uveitis seems to be associated with systemic disease, or idiopathic uveitis (*p* = 0.001), lower BCVA (*p* = 0.001), and a refractory course despite the treatment, while no significant association of CME with gender (*p* = 0.065) emerged. A single dexamethasone implant injection was reported to be effective in reducing CMT and resulted in a significant gain in visual acuity (AV) [34–44]. As described by Pleyer et al. [45], from our data analysis there was a significant reduction in CMT at M1 (*p* = 0.001), associated with an improvement in BCVA (*p* = 0.002). No significant difference was observed between the resolution of CME in noninfectious posterior uveitis with known cause (either not associated with systemic disease, and those associated with systemic disease) compared to noninfectious posterior uveitis of idiopathic origin (*p* = 0.087). On the other hand, the CME has reappeared over time in a significant percentage of cases, in 37.5% after 4-month, and 16.1% after 6-month. For instance, Nobre-Cardoso et al. [46] documented the reappearance of CME in 31.3% of cases treated after 3-month, and Khurana et al. [15] described a recurrence of CME after the 6-month in 65% of cases [10]. Possible serious complications of chronic uveitis, associated or not to CME, such as macular ischemia, epiretinal membranes (ERM), and macular holes were happened [47].

In turn, the presence of ERM has a negative correlation with lower visual acuity and CME relapsing [33]. CMT was very thick (> 300 µm) at inclusion and significantly reduced after the 1-month DEX-implant (*p* = 0.001). Only the cystoid form of uveitic ME was included in the study, which is the most difficult entity to resolve [33]. The presence of ERM was associated with CME in a certain percentage of patients (35.7%). Of these, uveitis-related CME recurrence despite the DEX-implant occurred in 32.1% of cases of ERM associated. Regarding the persistence, or the recurrence of uveitis-related CME, it was hypothesized the microstructural disruption of the inner and outer blood-retinal-barrier as the result of the release of inflammatory cytokines [48–50]. It has already been revealed that the possibility of anatomical and functional modifications of the retinal capillary network can be negatively correlated with the CME recurrence, but it has been demonstrated in diabetic patients [26]. To our knowledge, no other studies in the literature estimated the microvascular changes of the retinal capillaries in CME posterior noninfectious uveitis after the DEX-implant have been found. Most of the studies, as seen, were based on follow-up through OCT, widely used in clinical practice. Although OCT has dramatically transformed the understanding and management of uveitis-related CME, it does not allow to evaluate the retinal microvascular characteristics, which could be the cause of the recurrence of CME in uveitis patients [51]. OCTA previously has proven being an interesting imaging tool in diagnosis, and management of retinal vasculitis [52–54], and choriocapillaritis [55, 56], as it allowed to visualize in detail the retinal [11] microvascular changes, which can be so easily assessed and quantified, to accurately identify the area of the FAZ [57], or the parafoveal capillary telangiectasia and shunting vessels [58], or the rarefaction of the perifoveal capillary network [59]. The current study suggests use of OCTA among the imaging techniques for identifying microvascular changes during the course treatment with DEX-implant in noninfectious posterior uveitis, whereas the other instruments fail to detect the retinal capillary plexuses. Although the complete intraretinal and subretinal fluid resorption observed though OCT images after DEX-implant, some microvascular anatomical and functional changes were revealed by OCTA findings. Our investigation showed a reduction in SVP measurements already within 2-month (84%), reaching 96.4% for up 1-year, however displaying an irregular profile in 69.6% of cases, persisting for up 1-year. The relapsing uveitis-related CME eyes with irregular superficial FAZ profile were in 51%, while the SVP measurements reestablished in 100% of cases. Conversely, the DVP parameters restored in a lower number of eyes within the 2-month (39.3%), remaining abnormal in 46.4% of cases for up 1-year. Despite DVP restored in 53.6% of cases for up 1 year, a capillary rarefaction ring around the FAZ appeared in 80.4% of cases. The relapsing uveitis-related CME eyes with abnormal DVP parameters were present in 41%, of which 92.1% showed a rarefaction ring had abnormal DVP. Enlarged deep FAZ was found in patients with posterior uveitis, both in the presence and absence of ME [60]. Significant changes in DVP parameters were previously detected in uveitis-related CME, matching with the site of intraretinal cystoid spaces in the inner retina (inner nuclear and plexiform layers) [61]. The enlarged deep FAZ coupled with the rarefaction of the perifoveal capillary network was described in other ocular diseases as microstructural damage to the retinal barrier [62, 63]. Persistent damage of the retinal capillary layers, both of the superficial, and particularly of the deep plexuses, may further explain the reason of the relapsing uveitis-related CME.

### Limits

In using the OCTA of the patient with uveitis, we also encountered some difficulties to be taken into account, such as the possibility of the presence of synechiae, vitreous turbidity, dense cataracts, which may hinder good quality in image acquisition; to these limitations it is necessary to add age heterogeneity and patient collaboration, which were also crucial for a good quality of acquisition. Also, FAZ area was set manually. However, this study gives new insights in the potential fields of interest in future larger prospective clinical studies.

## Conclusions

Currently, OCT-A adds significant value to the multimodal imaging armamentarium in noninfectious posterior uveitis. It can be useful in monitoring complications such as the uveitis-related CME, and predictive of the relapsing CME. By embracing the hypothesis of the persistence of microvascular modifications of BRB in relapsing uveitis-related CME cases, the OCTA plays a decisive role to provide a microstructural analysis of the retinal capillary plexuses, representing a valid option for prognosis.

## Data Availability

The datasets during and/or analysed during the current study available from the corresponding author on reasonable request.
